# c-Jun-N-terminal phosphorylation regulates DNMT1 expression and genome wide methylation in gliomas

**DOI:** 10.18632/oncotarget.14330

**Published:** 2016-12-28

**Authors:** Dieter H Heiland, Roberto Ferrarese, Rainer Claus, Fangping Dai, Anie P Masilamani, Eva Kling, Astrid Weyerbrock, Teresia Kling, Sven Nelander, Maria S Carro

**Affiliations:** ^1^ Department of Neurosurgery, Medical Center – University of Freiburg, Freiburg, Germany; ^2^ Faculty of Medicine, University of Freiburg, Freiburg, Germany; ^3^ Department of Hematology, Oncology, and Stem Cell Transplantation, University of Freiburg Medical Center, Freiburg, Germany; ^4^ Department of Immunology, Genetics and Pathology and Science for Life Laboratories, University of Uppsala, Uppsala, Sweden

**Keywords:** Glioblastoma, G-CIMP, DNMT1, p-c-Jun, mesenchymal

## Abstract

High-grade gliomas (HGG) are the most common brain tumors, with an average survival time of 14 months. A glioma-CpG island methylator phenotype (G-CIMP), associated with better clinical outcome, has been described in low and high-grade gliomas. Mutation of *IDH1* is known to drive the G-CIMP status. In some cases, however, the hypermethylation phenotype is independent of *IDH1* mutation, suggesting the involvement of other mechanisms. Here, we demonstrate that DNMT1 expression is higher in low-grade gliomas compared to glioblastomas and correlates with phosphorylated c-Jun. We show that phospho-c-Jun binds to the DNMT1 promoter and causes DNA hypermethylation. Phospho-c-Jun activation by Anisomycin treatment in primary glioblastoma-derived cells attenuates the aggressive features of mesenchymal glioblastomas and leads to promoter methylation and downregulation of key mesenchymal genes (CD44, MMP9 and CHI3L1). Our findings suggest that phospho-c-Jun activates an important regulatory mechanism to control DNMT1 expression and regulate global DNA methylation in Glioblastoma.

## INTRODUCTION

High-grade gliomas are the most common brain tumors and are characterized by poor clinical outcome with an average survival time of 14 months (glioblastoma, WHO grade IV) [1]. In the past few years, glioblastoma subgroups characterized by specific gene signatures and associated with variable clinical outcome and survival have been described [2, 3]. In particular, the mesenchymal and the proneural groups appear as the two most robust subclasses identified in both studies [2, 3].

Epigenetic gene regulation has emerged as an important mechanism controlling gene expression in low and high-grade gliomas [4–7]. DNA methylation profiling of a large set of gliomas identified a subset of samples displaying concerted hypermethylation at a large number of loci, indicating the existence of a glioma-CpG island methylator phenotype (G-CIMP) [5, 7–9]. The G-CIMP tumors belong to the proneural subgroup and are associated with longer survival and better clinical outcome [5]. Although initially identified in a subset of high-grade gliomas, more extensive analysis showed that G-CIMP mostly characterizes low-grade tumors and confers improved survival [5, 6]. More recently, Turcan and colleagues identified *IDH1* mutation as a genetic event responsible for the establishment of the G-CIMP phenotype through DNA methylation remodeling [6]. Mechanistically, *IDH1* mutation induces accumulation of histone alterations such as H3K9me2, H3K27me3 and H3K36me3 which in turn promote DNA methylation [6]. Recently, it has been shown that *IDH1* mutation causes disruption of chromosome topology leading to aberrant oncogene activation [10].

The DNA methylatransferase-1 (DNMT1) enzyme is the principal maintenance DNA methyltransferase in human cancer cells [11], although cooperation of DNMT1 and DNMT3B is necessary for gene silencing. [12]. Additional reports also suggest a partial role of DNMT1 in establishing de novo methylation [13–15]. The enhanced expression of DNMT1 is responsible for change in the methylation patterns of tumor suppressor genes in cancer [16–18]. Moreover, increased expression of DNMT1 and DNMT3B was recently described in glioblastoma [19].

c-Jun is a basic leucine zipper (bZIP) transcription factor that acts as homo- or heterodimer, binding to DNA and regulating gene transcription, as part of the activator protein-1 (AP-1) complex [20]. Extracellular signals can induce post-translational modifications of c-Jun, resulting in altered transcriptional activity and target gene expression. This activates a number of cellular processes such as proliferation, apoptosis, survival, tumorigenesis and tissue morphogenesis [20, 21]. The transcriptional activity of c-Jun is regulated by environmental stress and cytokine-activated MAPK subfamilies which include ERK1/2, JNK and p38. JNK and p38 are the two kinases predominantly phosphorylating Jun [22, 23], although phosphorylation by ERK has been also reported in certain cells [24].

Here, we provide evidence for the first time that c-Jun N-terminal phosphorylation regulates DNMT1 expression in lower grade gliomas and proneural glioblastoma and promotes a global gene methylation profile similar to the G-CIMP phenotype. Our data suggest the existence of a c-Jun/DNMT1 pathway that functions as a regulator of global methylation in gliomas.

## RESULTS

### DNMT1 expression is increased in low-grade gliomas and is associated with improved survival

**Figure 1:**
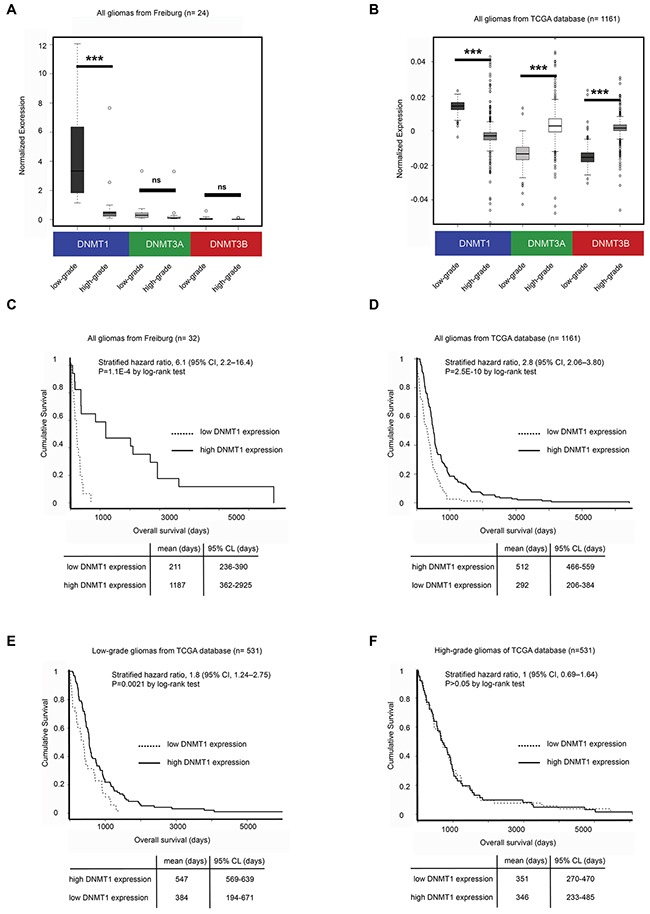
DNMT1 expression is high in low-grade gliomas and is associated with improved survival and global DNA methylation **A**. qRT-PCR Analysis of DNMT1, DNMT3A and DNMT3B expression in high-grade and low-grade gliomas from patient specimens collected at the University Medical Center Freiburg. **B**. Microarray analysis of DNMT1, DNMT3A and DNMT3B expression in tumor samples of high-grade and low-grade gliomas from the TCGA database. **C**. Kaplan-Maier and Cox regression analysis of glioma samples from Freiburg. **D**. Kaplan-Maier and Cox regression analysis of all gliomas from the TCGA database. **E**. Kaplan-Maier and Cox regression analysis of low-grade gliomas from the TCGA database. **F**. Kaplan-Maier and Cox regression analysis of high-grade gliomas from the TCGA database.

To study the role of DNMTs in gliomas, we used q-RT PCR to analyze the expression of the three DNA methyltransferase enzymes (DNMT1, DNMT3A and DNMT3B) in a panel of low and high-grade gliomas (n=32) collected at the University Medical Center Freiburg (Figure 1A and Supplementary Table 1). The expression of DNMT1 was higher in low-grade gliomas compared to high-grade tumors (4.57 fold, p-value=0.00059), but no difference was observed in DNMT3A and DNMT3B expression. The association of DNMT1 expression and low-grade gliomas compared to high-grade tumors was further validated through analysis of available gene expression data from The Cancer Genome Atlas (TCGA) (n=1161; fold=1.54; p-value=4.5E-127) (Figure 1B), whereas DNMT3A and DNMT3B were more associated with high-grade tumors (DNMT3A p-value=2.2E-16, DNMT3B p-value=2.1E-15) (Figure 1B). We then asked whether DNMT1 expression could also be relevant to tumor prognosis. We analyzed DNMT1 expression and patient survival data in tumors collected from Freiburg and from TCGA and found that DNMT1 was associated with improved patient outcome when gliomas from different tumor grades were included (p-value=1.1E-4) (Figure 1C and 1D). In order to evaluate the role of DNMT1 in patient survival within the same category, we also analyzed DNMT1 expression and survival separately in low and high-grade tumors from TCGA and found that DNMT1 was associated with better prognosis in low-grade (p-value=0.0021) (Figure 1E) but not in high-grade gliomas (p-value=0.9) (Figure 1F), suggesting either that high-grade gliomas are more homogeneous in terms of DNMT1 expression compared to low-grade gliomas or that other mechanisms could be involved.

### DNMT1 expression correlates with high DNA methylation

**Figure 2:**
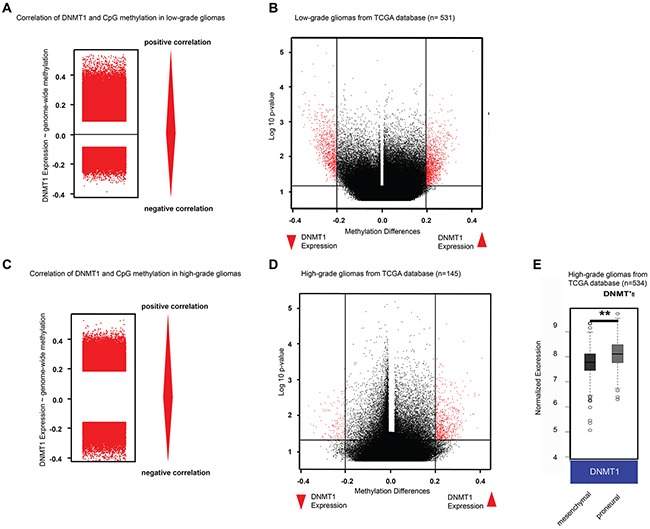
DNMT1 expression is associated with high global DNA methylation in gliomas **A**. Correlation analysis of DNMT1 expression and DNA methylation in low-grade gliomas. **B**. Volcano plot of low-grade gliomas grouped in high vs low *DNMT1* expression. **C**. Correlation analysis of DNMT1 expression and DNA methylation in high-grade gliomas. **D**. Volcano plot of high-grade gliomas grouped in high vs low *DNMT1* expression. **E**. Microarray analysis of DNMT1 expression in tumor samples of high-grade gliomas from the TCGA database.

Since low-grade gliomas are often characterized by the glioma-CpG island methylator phenotype (G-CIMP) [6], the high DNMT1 expression level in this tumor group could suggest a causal link to DNA methylation. To test this hypothesis, we looked at the association between DNMT1 expression and DNA methylation in low-grade and high-grade glioma samples from TCGA. Interestingly, DNMT1 expression was significantly correlated with overall CpG methylation level (Spearman-Ranked correlation and Fisher's Extract test for significance niveau p<0.05) (Figure 2A) in low-grade gliomas. Analysis of different methylated CpGs in low-grade tumors, represented as a volcano plot, shows a significantly increased (pairwise comparison t-test) methylation (red) in the group with high DNMT1 expression and vice versa (Figure 2B). A positive correlation with DNA methylation was also observed in high-grade gliomas, although to a lesser extent (Spearman ranked correlation and Fisher's Extract test for significance niveau p<0.05) (Figure 2C and 2D). In a manner similar to what we observed in the survival analysis (Figure 1F), this result could be affected by the underrepresented highly-methylated high-grade tumor samples. Finally, DNMT1 expression analysis in TCGA high-grade gliomas classified as mesenchymal and proneural showed a higher expression in proneural samples (mesenchymal 7.5±0.3; proneural 8.1±0.4, p=5.94E-11), consistent with previous findings indicating that G-CIMP glioma mostly belong to the proneural subtype (Figure 2E).

### Phosphorylated c-Jun regulates DNMT1 expression

**Figure 3:**
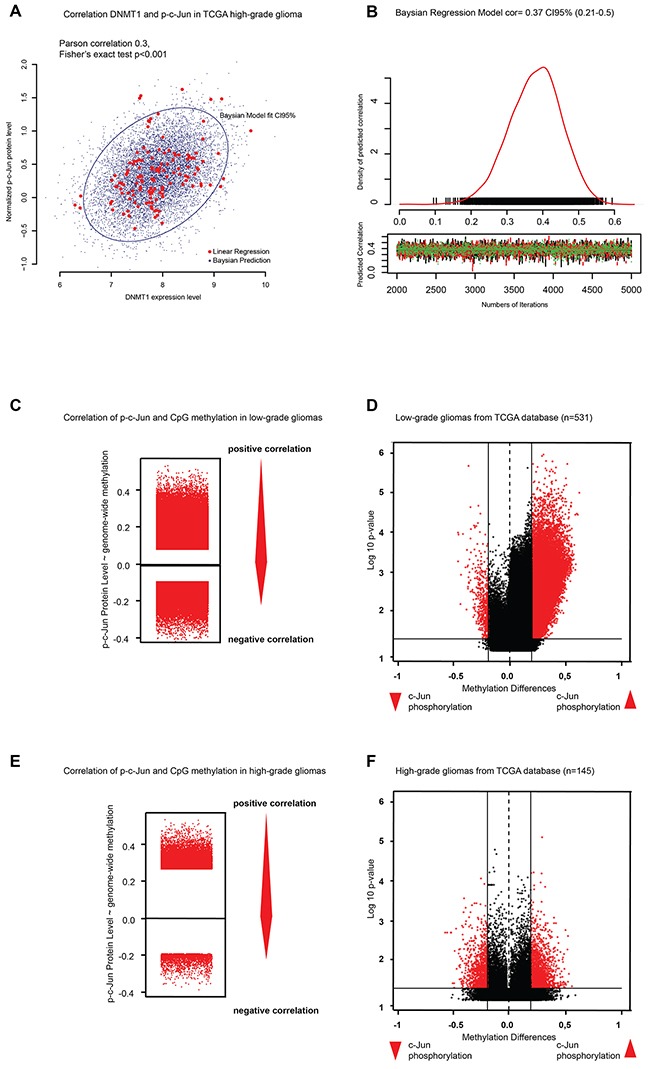
c-Jun phosphorylation correlates with DNMT1 expression and genome-wide methylation **A-B**. Correlation analysis of DNMT1 expression and c-Jun phosphorylation levels. **C**. Stripchart plot showing correlation between p-c-Jun and CpG methylation level in low-grade gliomas. **D**. Volcano plot showing different methylation of high vs. low phosphorylated c-Jun in low-grade gliomas. **E**. Stripchart plot showing correlation between p-c-Jun and CpG methylation levels in high-grade gliomas. **F**. Volcano plot showing differential DNA methylation in high-grade gliomas with high vs. low phosphorylated c-Jun.

A role of c-Jun in the regulation of DNMT1 has been previously suggested [25–27]. Moreover, c-Jun has been shown to prevent methylation at the *CDK6* promoter [28, 29]. Based on these earlier studies, we decided to look at the relationship between p-c-Jun, DNMT1 and global DNA methylation. Interestingly, we observed a significant correlation between DNMT1 RNA levels and p-c-Jun in high grade gliomas from TCGA (n=531, Pearson correlation=0.3, Baysian predicted correlation R=0.37 (95%CI 0.21-0.5), Fischer's exact test p= 8,9 x10^-5^) (Figure 3A–3B). Moreover, in the majority of the low-grade gliomas from TCGA samples analyzed, high p-c-Jun was also associated with high CpG methylation, suggesting a possible mechanistic link between p-c-Jun, DNMT1 and G-CIMP (Spearman's ranked correlation between p-c-Jun protein level and CpG methylation and Fisher's exact test for significance niveau p<0.05) (Figure 3C and 3D). A similar result was observed when high-grade gliomas were analyzed (Figure 3E and 3F). The observed effect of differential methylation was not as strong as in low-grade tumors but a high number of methylated CpG sites were significantly correlated to p-c-Jun level (Figure 3E).

**Figure 4:**
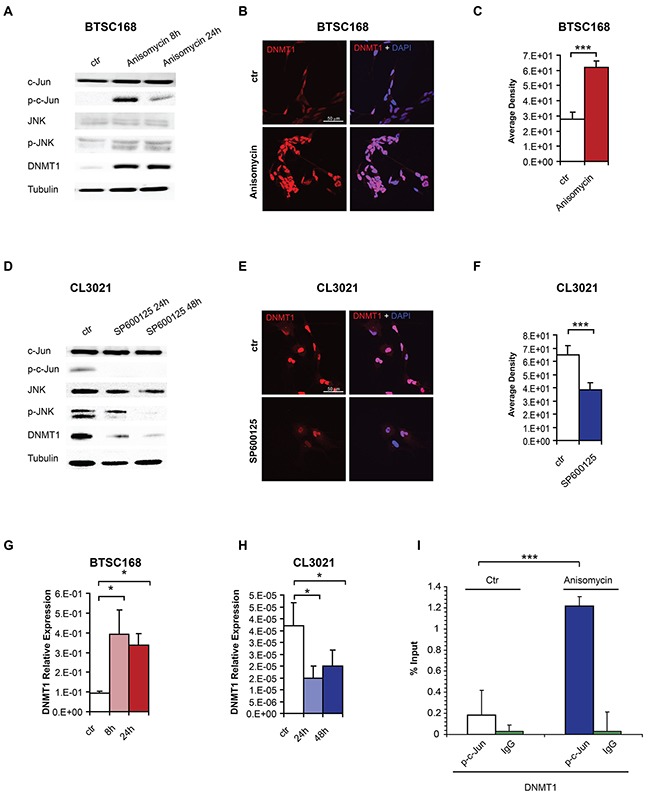
c-Jun phosphorylation regulates DNMT1 expression by promoter binding **A**. Immunoblot analysis of Jun, p-c-Jun, JNK, p-JNK, DNMT1 and alpha-tubulin (loading control) expression in a primary mesenchymal cell line (BTSC168) treated with Anisomycin as indicated. **B**. Immunofluorescence analysis of DNMT1 in BTSC168 after Anisomycin treatment. **C**. Quantification of immunostaining shown in B. **D**. JUN, p-Jun, JNK, p-JNK, DNMT1 and alpha-tubulin expression in a primary proneural cell line (CL3021) treated with JNK inhibitor SP600125 as indicated. **E**. Immunofluorescence analysis of DNMT1 in CL3021 after treatment with SP600125. **F**. Quantification of immunostaining shown in E. **G**. qRT-PCR of DNMT1 expression after JNK activation in BTSC168. Expression was normalized relative to 18s RNA. **H**. qRT-PCR of DNMT1 expression in CL3021 after JNK inhibition. Expression was normalized relative to 18s RNA. **I**. Plot of ChIP results after JNK activation in BTSC 168 cells. Significance level was defined as *p < 0.05, ***p < 0.005. Error bars represent mean ± standard error of the mean (SEM) of at least three independent experiments.

We then sought to validate the relationship between c-Jun phosphorylation and DNMT1 in patient-derived glioblastoma cells. Since no LGG-derived cells with characterized DNA methylation levels were available, we used glioblastoma-derived mesenchymal and proneural cell lines, since these two subtypes are usually G-CIMP- and G-CIMP+ respectively. Cell treatment with the p-c-Jun activator Anisomycin [30] in the mesenchymal cell line BTSC168 resulted in increased JNK and c-Jun phosphorylation and DNMT1 protein expression (Figure 4A). We noticed that p-c-Jun was higher after 8 hours and subsequently decreased, while DNMT1 levels were more stable, suggesting that other transcription factors and/or co-factors could sustain DNMT1 expression in the absence of p-c-Jun at later time points. Immunostaining also confirmed the increased DNMT1 protein levels (Figure 4B and 4C). Given the broad spectrum of cell activity by Anisomycin, we sought to confirm our findings by using a JNK inhibitor. Inhibition of c-Jun phosphorylation by the JNK inhibitor SP600125 [31] in a proneural cell line (CL3021) expressing high levels of p-c-Jun reduced JNK and c-Jun phosphorylation (Figure 4D) and DNMT1 protein levels (Figure 4D-4F). qRT-PCR analysis confirmed an effect of c-Jun phosphorylation on DNMT1 RNA levels upon Anisomycin or SP600125 treatment in BTSC168 and CL3021 cells respectively (Figure 4G and 4H). We then performed chromatin immunoprecipitation to test binding of p-c-Jun at the *DNMT1* promoter. Treatment of BTSC168 with Anisomycin resulted in increased precipitation of the *DNMT1* promoter, indicating that p-c-Jun binds to the *DNMT1* promoter and regulates its expression (Figure 4I).

### Genome-wide methylation is influenced by c-Jun phosphorylation status

**Figure 5:**
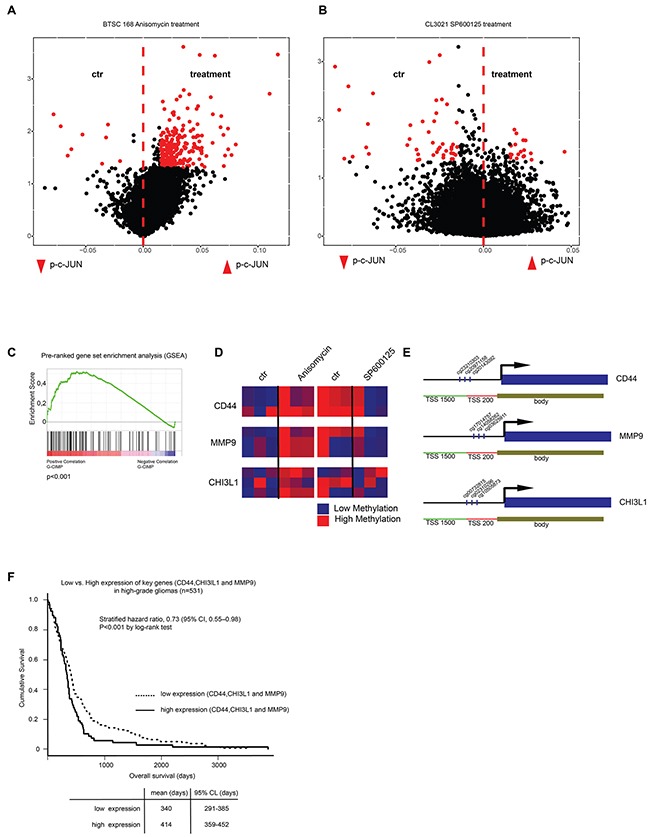
C-Jun phosphorylation induces genome wide methylation **A-B**. Volcano plots showing results of 450k methylation array of Anisomycin-treated BTSC168 cells (A) and SP600125-treated CL3021 cells (B). **C**. Gene Set Enrichment Analysis of differentially-methylated promotors after Anisomycin treatment (p=0.001). **D**. Heatmap showing methylation changes at the promoter of candidate genes (*CD44*, *MMP9*, *CHI3L1*) in Anisomycin-treated BTSC168 cells (left panel) and SP600125-treated CL3021 cells (right panel). **E**. Schematic representation of *CD44*, *MMP9*, *CHI3L1* gene loci. The position of CpG regions analyzed in (B) is indicated. **F**. Kaplan-Maier and Cox regression analysis of high-grade gliomas from the TCGA database split into low key-gene expression vs. high key-gene expression (20% highest v. lowest).

We then asked whether c-Jun phosphorylation is involved in the establishment of global DNA methylation in gliomas through regulation of DNMT1 expression. To test our hypothesis, cells with active (BTSC168) or inhibited (CL3021) JNK pathway were used to measure genome-wide DNA methylation changes by 450k methylation array. Anisomycin-treated BTSC168 cells revealed a significant increase in genome-wide DNA methylation of promoter regions in comparison to untreated cells (Figure 5A). Conversely, global DNA methylation was reduced in CL3021 cells treated with JNK inhibitor SP600125 (Figure 5B). In order to understand whether the differentially-methylated loci overlap with the previously reported G-CIMP signature, we performed a gene-set enrichment analysis and discovered a significant (p<0.0001) enrichment of methylation in the G-CIMP gene-set corresponding to c-Jun phosphorylation (Figure 5C). Interestingly, highly methylated loci in the G-CIMP signature were also highly methylated in BTSC168 upon treatment (Figure 5C). This suggests that p-c-Jun causes global methylation changes in glioblastoma and low-grade gliomas similar to those of G-CIMP by regulating DNMT1 expression.

### Phospho-c-Jun epigenetically regulates the expression of mesenchymal signature genes

**Figure 6:**
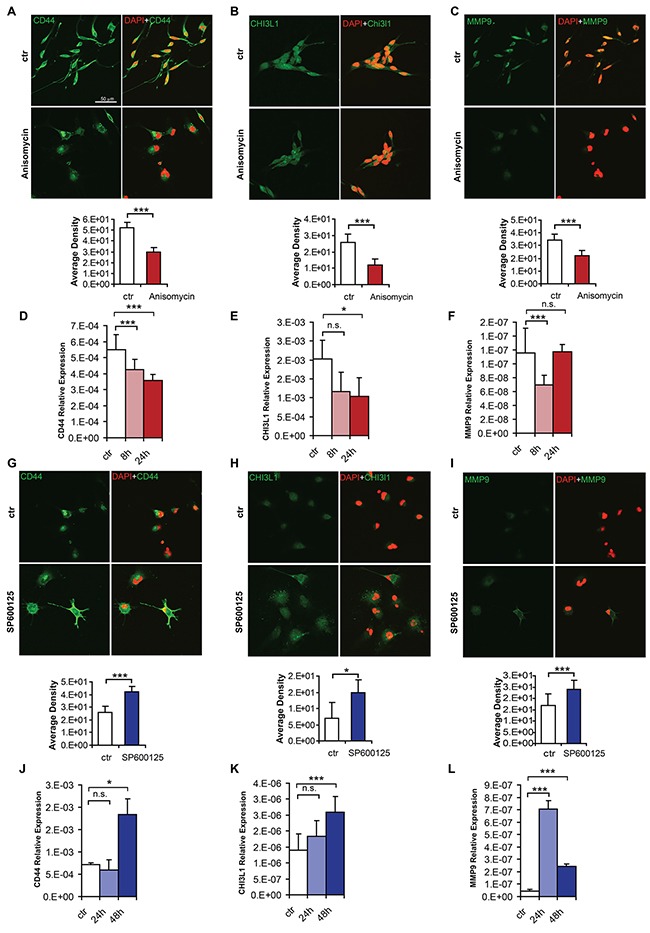
C-Jun phosphorylation regulates expression of key mesenchymal genes **A-C**. Immunofluorescence analysis of CD44 (A), CHI3L1 (B) and MMP9 (C) expression upon Anisomycin treatment in BTSC168 cells (top panels). The scale bar represents 50 μm. Bottom panels: quantification of CD44, CHI3L1 and MMP9 immunostaining. **D-F**. qRT-PCR analysis of CD44 (D), CHI3L1 (E) and MMP9 (F) expression in BTSC168 treated with Anisomycin. **G-I**. Immunofluorescence analysis of CD44 (G), CHI3L1 (H) and MMP9 (I) expression upon treatment with JNK inhibitor SP600125 in CL3021 cells. The scale bar represents 50 μm. Bottom panels show corresponding quantification. **J-L**. qRT-PCR analysis of CD44 (J), CHI3L1 (K) and MMP9 (L) expression in CL3021 treated with SP600125. Significance level was defined as *p < 0.05, ***p < 0.005. Error bars represent mean ± standard error of the mean (SEM) of at least three independent experiments.

Promoter methylation of three key mesenchymal genes (*CD44*, *MMP9* and *CHI3L1*) already known to be regulated by DNA methylation [5, 6, 32] appeared to be modified by p-c-Jun activation or inhibition (Figure 5D and 5E). This suggests that the loss of mesenchymal properties could be a consequence of DNA methylation changes. Consistent with a role of CD44, MMP9 and CHI3L1 in aggressive glioblastoma properties, low expression of the three genes was associated with better outcome in TCGA samples (Figure 5F). To confirm that the epigenetic changes induced by activation or inhibition of c-Jun phosphorylation result in mesenchymal gene expression changes, protein and RNA levels of CD44, MMP9 and CHI3L1 were analyzed. Immunostaining of BTSC168 cells treated with Anisomycin showed a decrease in protein level for all genes tested (Figure 6A–6C, top and bottom panels). Concordantly, CD44 and CHI3L1 mRNA levels were significantly decreased after 24h (p=0.00003 and p=0.03 respectively) (Figure 6D and 6E) while expression of MMP9 was only transiently reduced at 8h (p=0.0003) (Figure 6F). Treatment with the JNK inhibitor SP600125 produced an opposite effect as immunostaining analysis revealed an increased level of CD44, MMP9 and CHI3L1 protein (Figure 6G–6I, top and bottom panels). q-RT-PCR showed an increase of CD44 and CHI3L1 mRNA levels after 48h (p=0.01 and p=0.007 respectively) (Figure 6J–6K) while MMP9 expression was significantly upregulated after 24h (p=0.002) and to a lesser extent after 48h (p=0.003) (Figure 6L). These data indicate that JNK-pathway-dependent modifications of c-Jun phosphorylation status produce epigenetic alterations which affect gene expression of key mesenchymal genes.

Previous studies indicate that NF-kB signaling could be regulated by c-Jun activation [33–36]. To rule out whether DNMT1 could be involved in this mechanism, we analyzed NF-kB activity by measuring nuclear binding of the canonical p65 NF-kB subunit to a kB-responsive sequence. Interestingly, Anisomycin treatment led to an increase in p65 binding indicating that c-Jun phosphorylation activates NF-kB signaling, consistent with previous studies (Supplementary Figure 1C). However, DNMT1 silencing did not affect NF-kB activity suggesting that other factors downstream of c-Jun could be implicated (Supplementary Figure 1C). Moreover, DNMT1 reduction was associated with higher CHI3L1 protein levels, consistent with a decrease in DNMT1-mediated epigenetic regulation (Supplementary Figure 1A and 1B). Interestingly, Anisomycin treatment did not rescue DNMT1 knockdown effect indicating that DNMT1 is required for CHI3L1 transcriptional regulation (Supplementary Figure 1A and 1B).

### c-Jun N-terminal phosphorylation affects cell migration and invasion

**Figure 7:**
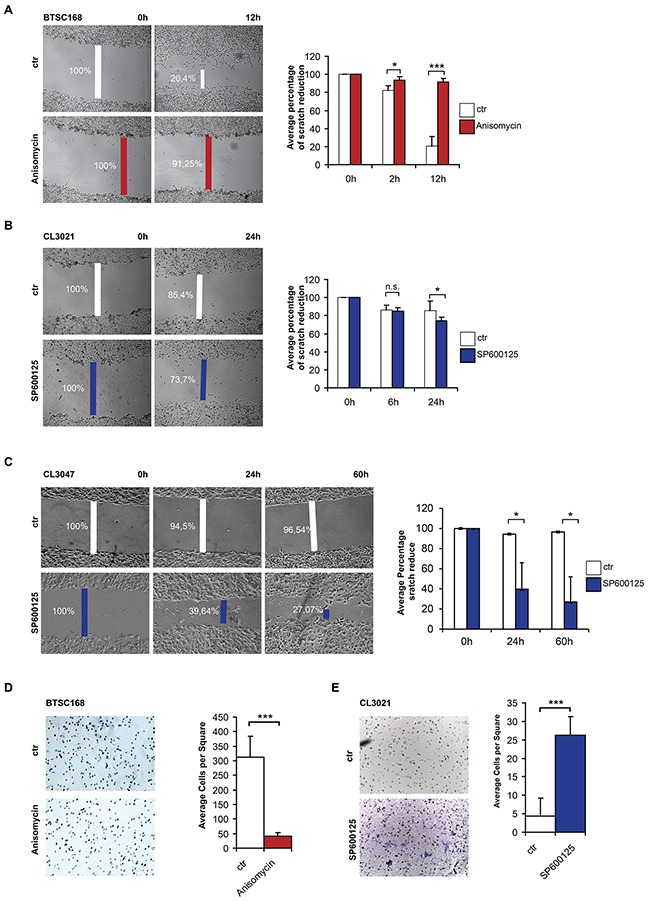
C-Jun phosphorylation reduces cell migration and invasion **A**. Microphotographs showing migration of BTSC 168 cells with or without Anisomycin treatment in a scratch assay experiment. Quantification of each experiment is shown in the corresponding right panel. **B-C**. Scratch assay of CL3021 (B) and CL3047(C) cells after JNK inhibition. Characterization of CL3047 cells is shown in Supplementary Figure 1. Quantification of each experiment is shown in the corresponding right panel. **D**. Microphotographs showing invading BTSC168 cells upon JNK activation (left panel) and corresponding quantification (right panel). **E**. Microphotographs showing invading CL3021 cells upon JNK inhibition (left panel) and corresponding quantification (right panel). Significance level was defined as *p < 0.05, ***p < 0.005. Error bars represent mean ± standard error of the mean (SEM) of at least three independent experiments.

Our data indicate that increased levels of c-Jun phosphorylation and subsequent induction of DNMT1 expression are responsible for the G-CIMP/proneural phenotype of glioma and adverse to the mesenchymal phenotype. Given that mesenchymal glioblastoma are characterized by aggressive features such as invasion and migration, we analyzed the role of p-c-Jun in such cell properties. Migratory properties of JNK-activated and inhibited cells was tested via scratch assay. Treatment of BTSC168 with Anisomycin resulted in a strong inhibition of cell migration compared to controls (Figure 7A). In contrast, treatment of CL3021 increased the percentage of space covered by the cells (85% vs 70%) after 24h (Figure 7B). The result was even more pronounced when a second proneural cell line (CL3047) was used (96.5% vs 27%) (Figure 7C and Supplementary Figure 2). Measurement of cell invasion in BTSC168 treated with Anisomycin showed a significant (p=6,8E-15) reduction of invasion compared to the control group (Figure 7D). Instead, treatment with the JNK inhibitor SP600125 in proneural CL3021 cells significantly increased cell invasion compared to the control (p=1,6E-12) (Figure 7E), indicating that inhibition of c-Jun phosphorylation by JNK inhibitor increases cell invasion.

## DISCUSSION

In this study, we have described a new pathway involved in the establishment of global DNA methylation profile resembling that of the previously identified G-CIMP, a molecular signature associated with improved survival in low and high-grade gliomas. We demonstrate that highly methylated gliomas contain high levels of p-c-Jun compared to sparsely methylated gliomas. Interestingly, this pattern correlates with DNMT1 expression, suggesting a relationship and a regulatory mechanism between the two genes. Activation of p-c-Jun in a primary mesenchymal cell line leads to increased DNMT1 expression and attenuates the aggressive features of glioma cells. In addition, we show that increased c-Jun phosphorylation leads to changes in global DNA methylation patterns towards one that is similar to the G-CIMP with concomitant downregulation of key mesenchymal genes.

The G-CIMP was initially reported in glioblastoma and associated with the proneural signature, although additional analysis integrating lower grade gliomas revealed that this phenotype is prevalent in low-grade tumors and confers improved survival [5, 6, 8]. It was demonstrated the *IDH1* mutation leads to the establishment of the G-CIMP [6, 10]. However, in some cases the hypermethylation phenotype also occurs in the absence of *IDH1* mutations, suggesting that other mechanisms could be involved.

Our analysis of p-c-Jun and DNMT1 expression in clinical glioma biopsy tissues from TCGA highlighted a good correlation between p-c-Jun and DNMT1, supporting previous results in nasopharyngeal carcinoma indicating a role of p-c-Jun in DNMT1 regulation [26]. Consistent with our data, Tsai and colleagues demonstrated that p-c-Jun binds to a *DNMT1* promoter inducing DNMT1 expression and subsequent methylation of the *CDH1* (a.k.a. E-Cadherin) gene. Although only one gene promoter was analyzed, it cannot be excluded that p-c-Jun and consequent DNMT1 regulation in nasopharyngeal carcinoma might affect methylation of a larger set of genes. A different mechanism, still suggesting a connection between c-Jun and DNA methylation, describes a new role of c-Jun as a protective factor that prevents DNA from being methylated [28, 29]. So, according to our model, N-terminal phosphorylation of c-Jun could mediate a switch from unmethylated to methylated DNA. A recent report indicates that c-Jun/c-Fos heterodimers bind to methylated AP1 sites in the genome and activate transcription [37]. Although this c-Jun-mediated function seems to be in contrast with our reported role of c-Jun in mediating methylation, it is possible that c-Jun can selectively regulate the expression of a subset of genes containing AP1 sites in the promoter.

We show that DNMT1 expression is lower in mesenchymal glioblastoma compared to proneural tumors. However, since correlation analysis between DNMT1 and CpG methylation in high-grade tumors does not distinguish between tumor subclasses, it is possible that tumors from the classical or neural subtype might also consistently show high or low levels of DNMT1. Nevertheless, since the highly methylated glioblastoma (G-CIMP) are mostly proneural [5], we would mostly expect association between DNMT1 expression and DNA methylation in proneural samples. In the future, more analysis would be necessary to rule out whether an association between DNMT1, CpG methylation and the other subclasses exists.

We show that activation of p-c-Jun is associated with increased methylation and consequent downregulation of three mesenchymal genes which have been previously described as key genes involved in the aggressive features of mesenchymal glioblastomas [32, 38]. Since mesenchymal gliomas are mostly G-CIMP-negative and have been identified as belonging to a molecular subclass that is mutually exclusive to the often G-CIMP-positive proneural tumors, it is possible that methylation and consequent downregulation of many mesenchymal genes in proneural gliomas is responsible for the improved outcome of these tumors. Consistent with this idea, we show that the increased methylation by p-c-Jun is associated with reduced cell migration and invasion, two hallmarks of aggressive gliomas.

Our proposed role of p-c-Jun as a mediator of a G-CIMP-like phenotype in glioblastoma differs from previous reports showing that levels of c-Jun in gliomas correlate with the grade of malignancy and that this increase contributes to the malignant properties of the cells [39, 40]. Similarly, activation of JNK has been associated with increased self-renewal and tumor-initiating capacity of glioma stem cells [41–43]. Since our study focuses on association of DNMT1 and p-c-Jun with the G-CIMP/proneural subclass of glioblastoma, it is possible that our observed phenotype might specifically characterize this glioma subtype and would not be observed in the others. Nevertheless, additional studies would be required to clarify the role of JNK and c-Jun in low and high-grade gliomas.

In summary, we have demonstrated that phosphorylated c-Jun directly binds to and activates the *DNMT1* gene promoter and represents a novel regulatory module of genome-wide methylation status in glioblastoma and low-grade gliomas. Since G-CIMP tumors have been described as less aggressive and characterized by a better outcome, the c-Jun/DNMT1 pathway could potentially serve as a new target for the treatment of glioblastoma.

## MATERIALS AND METHODS

### Tumor samples and patients

Glioblastoma samples from patients were collected at the Department of Neurosurgery, University Medical Center Freiburg, Germany. Retrieval and scientific analysis of patient-derived tissue was approved by the local ethics committee under protocol 100020/09. Written informed consent was obtained from all patients. Further analysis was performed on 537 glioblastoma samples collected as part of The Cancer Genome Atlas (TCGA) Pilot Project ( http://cancergenome.nih.gov/).

### Cell lines and cell culture

The primary brain tumor stem cells (BTSCs) were prepared from tumor specimens collected at the University of Freiburg (BTSC168) or University of Uppsala (CL3021 and CL3047) and grown as neurospheres in Neurobasal medium (Invitrogen) containing B27 supplement (Invitrogen), FGF (20 ng/ml, R&D Systems), EGF (20 ng/ml, R&D Systems), LIF (10 ng/ml, Genaxxon biosciences), Heparin (2 µg/ml, Sigma) and glutamax (Invitrogen). For CL3021 and CL3047 culture, N2 supplement (Invitrogen) was added. To activate the JNK-pathway, 1μl/ml Anisomycin (Sigma Aldrich) was added to cell culture medium according to the manufacturer's instructions. For inactivation of the JNK-pathway, cells were treated with 50μmol/ml SP600125 (Invivogene) according to the manufacturer's instructions.

### Classification of brain tumor cells

The classification of BTSCs previously profiled by gene expression array (Illumina HumanHT-12v3) was performed using 510 genes out of the 840 classifier genes used by Verhaak et al. to classify 260 glioblastoma samples (Verhaak et al., 2010), and 529 glioblastoma tissue samples from TCGA with assigned subtypes as reference (Cancer Genome Atlas Research Network, 2008). The 510 genes were selected for their concordance with the extended set of 529 TCGA samples and representation on the Illumina HumanHT-12v3 expression BeadChip arrays. The expression levels for these genes on the Illumina arrays and in the TCGA data set were converted into z-scores and the combined matrix was used to classify each BTSC sample based on a k-nearest neighbors (k=10) and voting procedure, in which a subtype was assigned based on the majority subtype among the 10 TCGA samples with highest correlation coefficients for these genes with respect to the BTSC sample. All data manipulations were performed in R (R Core Team, 2012) and MATLAB (The MathWorks, Inc., Natick, MA, United States).

### Immunoblotting and immunostaining

The following antibodies were used in immunoblotting analyses: phospho-c-Jun (Ser73) (Cell Signaling dilution: 1:500), c-Jun (Cell Signaling dilution: 1:1000), DNMT1 (Abcam dilution: 1:1000), JNK (Santa Cruz, dilution: 1:5000), phospho-JNK (Santa Cruz, dilution: 1:5000), and α-tubulin (mouse monoclonal, Abcam). Primary antibodies were used at the concentration indicated by the manufacturers. Anti-Mouse and anti-Rabbit HRP-conjugated (Santa Cruz, dilution: 1:5000) were used as secondary antibodies. Immunostaining was performed using antibodies against DNMT1 (Abcam dilution: 1:200), CD44 (Abcam dilution: 1:200), CHI3L1 (Quidel dilution: 1:150) and MMP9 (Cell signaling dilution: 1:200). Primary antibodies were used at the concentration indicated by the manufacturers. Anti-Mouse, anti-Rabbit and anti-Goat Alexa594- or Alexa647-conjugated (Life Technologies) were used as secondary antibodies. Alexa594-conjugated antibodies were used at 1:200 dilution and Alexa647-conjugated antibodies were used at 1:100 dilution. Pictures were acquired using a fluorescent microscope (FL10i, Olympus).

### Quantitative real-time PCR

Total RNA was prepared using the RNeasy kit or the All Prep DNA/RNA Protein Mini Kit (Qiagen) and used for first strand cDNA synthesis using random primers and SuperscriptIII Reverse Transcriptase (Invitrogen). Quantitative real-time PCR (qRT-PCR) was performed using a SYBR Green PCR Master Kit (Applied Biosystems). The following primers were used: DNMT1-for: AGGCGGCTCAAAGATTTGGAA; DNMT1-rev: GCAGAAATTCGTGCAAGAGATTC; DNMT3A-for: GTCATGTGGTTCGGAGACGG; DNMT3A-rev: AGT GTCACTCTCATCGCTGTC; DNMT3B-for: CCCAGCT CTTACCTTACCATCG; DNMT3B-rev: GGTCCCCTAT TCCAAACTCCT; CD44-for: GCAACTGAGACAGCA ACCAAG; CD44-rev: GCCATTTGTGTTGTTGTGTG AA; CHI3L1-for: CCACCCTAATCAAGGAAATGA; CH I3L1-rev: TGAAATCCAGGTGTTGGGATA; MMP9- for: TGTACCGCTATGGTTACACTCG; MMP9-rev: GGCAGGGACAGTTGCTTCT; 18srRNA-for: TTTGCG AGTACTCAACACCA; 18srRNA-rev: CCACACCCCT TAATGGCA

### Migration and invasion assays

For the wound-healing assay, cells were plated in 60 mm dishes and grown at 95% confluence. A scratch of approximately 1 mm was made with a p1000 pipette tip and fresh medium containing 1μl/ml Anisomycin or water was added to the cells. Pictures were taken after 0h, 2h and 12h. For the JNK inactivation experiment, SP600125 or DMSO was added and pictures were taken after 0h, 6h and 24h. Images were taken using a wide-field microscope (Axiovert, Zeiss). The Matrigel invasion assay was performed using BioCoat Matrigel Invasion Chambers (BD Bioscience) according to the manufacturer's instructions. 8000 cells/well were seeded in the upper compartment and incubated with Anisomycin or SP600125. PDGF-BB (20ng/ml, R&D) was used as a chemoattractant. After the incubation, cells were fixed with formaldehyde and stained with crystal violet.

### Methylation array and gene set enrichment analysis (GSEA)

The methylation array was performed using the Illumina Infinium HumanMethylation450 chip according to the manufacturer's instructions (DKFZ, Heidelberg). Data analysis was performed by R software and RnBeads software package. Quality control was done by major quality control algorithms including sample-independent controls. Normalization of the raw data was done by SWAN and BMIQ procedures. Differential methylation on the promoter level was computed based on a variety of metrics. Differential methylation analysis was performed by limma package. Gene-set Enrichment Analysis (GSEA) was performed by GSEA tool ( http://software.broadinstitute.org/gsea/index.jsp). G-CIMP gene set was defined as a promoter set including 100 most differently methylated gene promoters as described in Noushmehr et al. (2010).

### Chromatin immunoprecipation

Chromatin immunoprecipitation (ChIP) was performed as previously described [44]. 4×10^7^ BTSC168 cells treated with Anisomycin (8h) or controls were fixed with 1% formaldehyde for 15 minutes and stopped with 0.125M glycine for 5 minutes and washed twice with PBS. Cells were harvested using SDS buffer with 1X Protease inhibitor and PMSF (Phenyl Methyl Sulfonyl Fluoride) and centrifuged at 1200rpm/6min at 4°C. The pellet was suspended in 3 ml of ice-cold immunoprecipitation buffer. Samples were sonicated and the sonicated lysates were precleared with Protein A/G beads (Santa Cruz) and incubated at 4°C overnight with 3 μg of polyclonal antibody specific for phospho c-jun (rabbit polyclonal, Cell Signalling) or normal rabbit immunoglobulins (Santa Cruz). DNA was eluted in 200 μl of water and the immunoprecipitated DNA was analyzed by absolute qRT-PCR. The amplification product was expressed as percentage of the input for each condition. The following primers were used to amplify sequences surroundings predicted c-Jun binding sites at the DNMT1 locus: DNMT1_forward: GAAAGTTTAAGGCCGGGCAC, DNMT1_reverse: GATCACTGC-AGCCTCTACCT.

### Vectors and lentiviral infection

Lentiviral infections were performed as previously described [45]. Knockdown of DNMT1 was obtained with a shRNA lentiviral vector (pLKO, Sigma Clone ID TRCN0000021891). Upon infection cells were selected with Puromycin (0.5 μl/ml) for 24 hours.

### Nuclear and cytoplasmic extracts and NF-kB DNA binding assay

Cytoplasmic and nuclear extracts were prepared using a Nuclear Extract Kit (Active Motif) and processed using the TransAM assay p65 kit according to the manufacturer´s instructions.

### Statistical analysis

Expression analysis of the TCGA data was performed by R software. TCGA samples were extracted from the TCGA database and lowess normalized. Expression fold change was calculated by the log2 ratios of intensities. Statistical significance was defined as p < 0.05 for all statistical tests. For methylation analysis of TCGA samples, level 3 methylation data (Illumina Infinium HumanMethylation450 chip) were extracted from the TCGA database and normalized. Analyses were done as described above. For survival analysis, the Kaplan-Meier method was used to provide median point estimates and time-specific rates. The ANOVA model was used in univariate and multivariate settings to identify significant factors associated with the treatment. Log-rank, Wilcoxon and Tarone-Ware tests were used to analyze survival parameters such as OS and PFS. The Hazard-Ratio (HR) was calculated by Cox-Regressions tests. Statistical significance was defined as p < 0.05 for all statistical tests. A regression model was used to correlate c-Jun phosphorylation and DNMT1 gene expression by maximum likelihood estimation (MLE). Additionally, a Baysian regression model was performed based on the Markov Chain Monte Carlo (MCMC) algorithm to calculate a probability distribution and predict unknown parameter. Both models showed a robust correlation. All statistical analyses were performed using R-software and R-software tools or IBM SPSS Statistics version 22. Plots were done by R-software package ggplot2.

## SUPPLEMENTARY MATERIALS FIGURES AND TABLE


