# Supplementary material for: c-Jun-N-terminal phosphorylation regulates DNMT1 expression and genome wide methylation in gliomas
Source: Oncotarget. 2016 Dec 28;8(4):6940–54. doi: 10.18632/oncotarget.14330 (PMC5351681; doi:10.18632/oncotarget.14330)
Supplement: Supplementary file 1 [file oncotarget-08-6940-s001.pdf]

c-Jun-N-terminal phosphorylation regulates DNMT1 expression and genome wide methylation in gliomas

SUPPLEMENTARY FIGURES AND TABLE

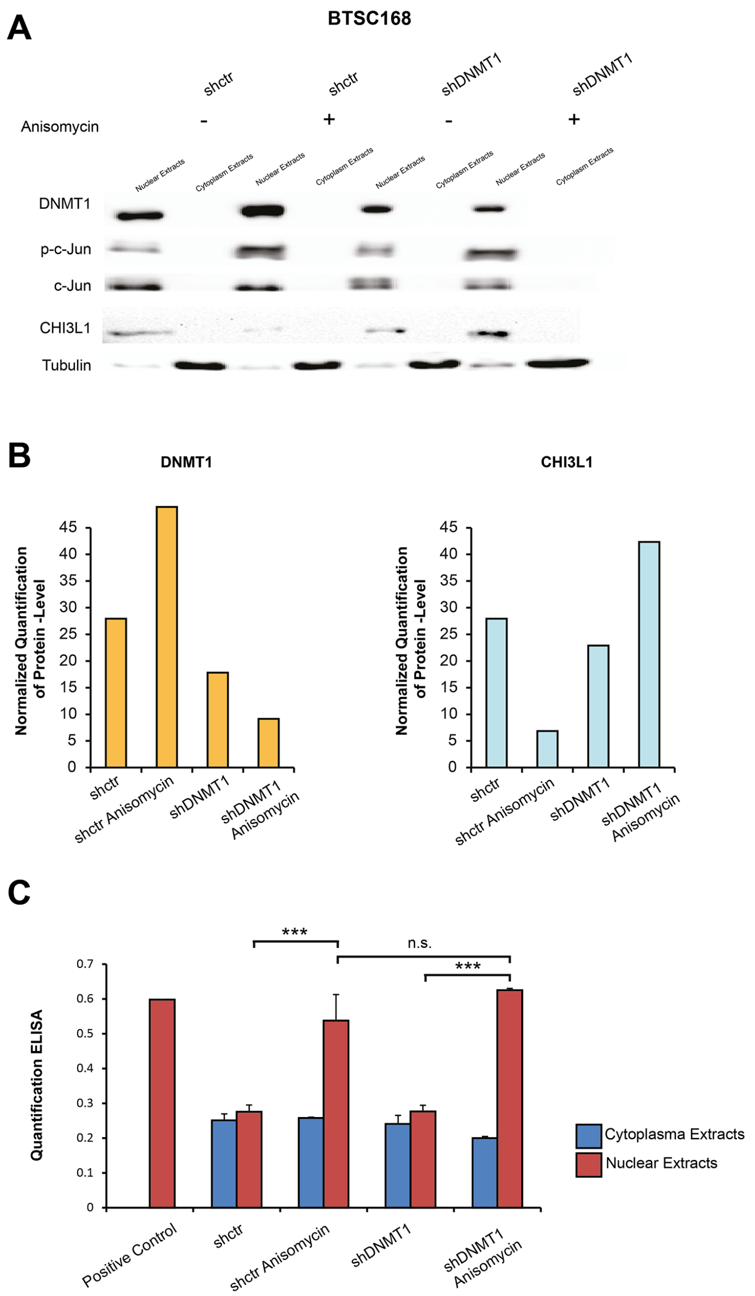

Supplementary Figure 1: NF-κB activation by p-Jun does not require DNMT1. A-B. Immunoblotting analysis of BTSC168 transduced with shctr or shDNMT1 lentivirus, and treated with Anisomycin. The quantification of DNMT1 and CHI3L1 was normalized based on the tubulin protein-level (B). **C.** Analysis of NF-κB nuclear binding to a κB-responsive sequence, in nuclear and cytoplasm extracts. Significance level was defined as $*p < 0.05$, $***p < 0.005$. Error bars represent mean ± standard error of the mean (SEM) of at least three independent experiments.

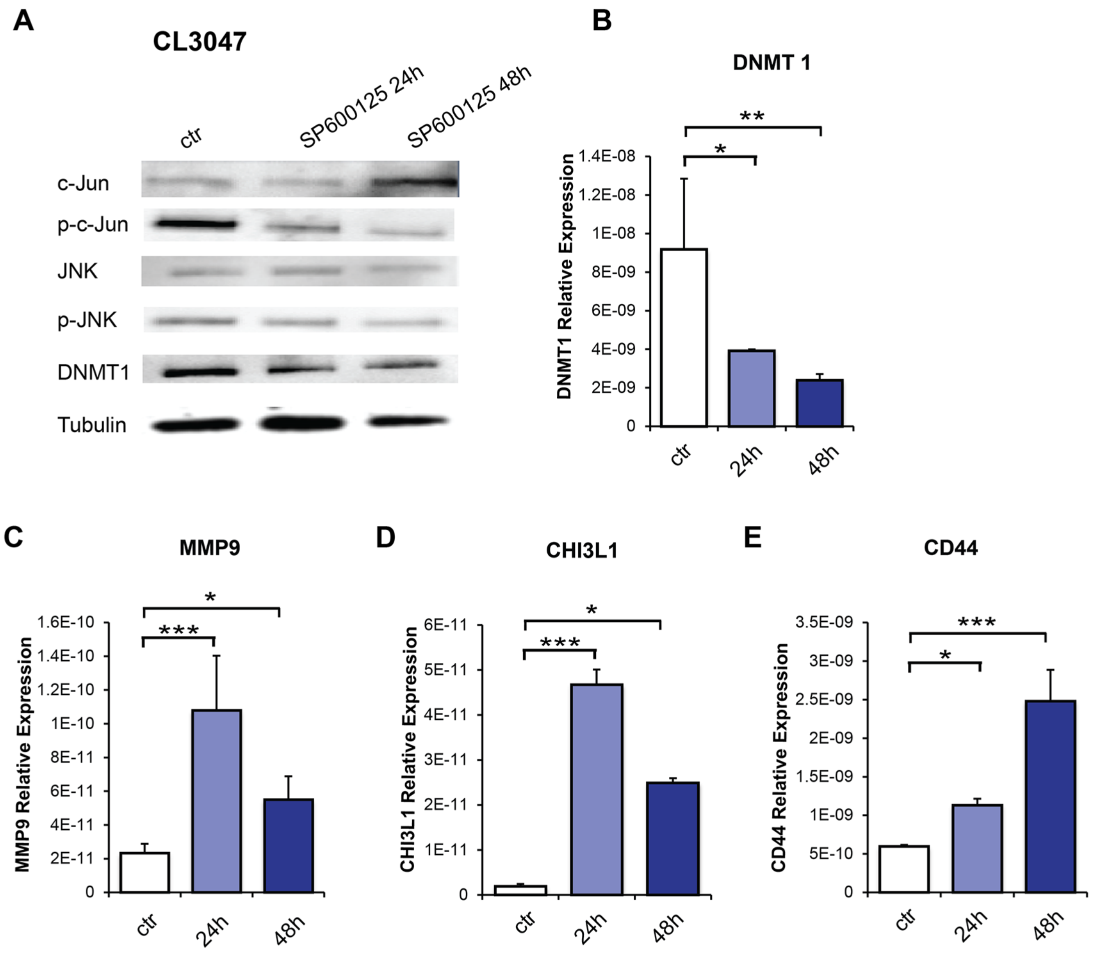

Supplementary Figure 2: Characterization of CL3047 cells. A. Immunoblot analysis of c-Jun, p-c-Jun, JNK, p-JNK and DNMT1 expression in the primary proneural cell line CL3047. **B.** q-RT PCR showing DNMT1 expression in CL3047 upon treatment with JNK inhibitor SP600125. C-D. q-RT PCR showing expression of the three mesenchymal genes (MMP9, CHI3L1 and CD44) in CL3021, upon treatment with JNK inhibitor SP600125.

Supplementary Table 1: Table of Freiburg glioma patients

Patient	DNMT1	Grade	Tumor	PFS	OS	Age
**BT178**	0.10223082	IV	GBM	2.9	118.3	75.5
**BT66**	0.10298547	IV	GBM	12.9	423.2	72.5
**BT175**	0.12928351	IV	GBM	1.9	153.4	73.7
**BT163**	0.1757574	IV	GBM	4.2	330.9	63.7
**BT25**	0.18325079	IV	GBM	5.6	202.6	72.4
**BT146**	0.28164113	IV	GBM	0.5	47.1	69.2
**BT143**	0.32415333	IV	GBM	6.6	231.6	73.1
**BT12**	0.33350049	IV	GBM	7.9	271.7	68.2
**BT10**	0.45983831	IV	GBM	9.4	316.9	73.8
**BT205**	0.48212423	IV	GBM	1.2	68.2	63.5
**BT48**	0.50130003	IV	GBM	0.7	52.1	79.3
**BT5**	0.50496325	IV	GBM	3.3	136.4	63.2
**BT197**	0.58272251	IV	GBM	5.9	210.6	63.9
**BT108**	0.9124834	IV	GBM	11.8	390.1	70.9
**BT206**	0.99113128	IV	GBM	3.0	687.9	54.2
**BT101**	1.14044974	II	O	93.9	6835.7	24.0
**BT280**	1.4295224	II	OA	68.0	1187.2	22.7
**BT187**	1.54053316	II	A	81.3	2925.0	38.7
**BT69**	1.81343056	III	AOA	3.5	137.4	76.0
**BT32**	1.84820793	III	AOA	9.7	6835.7	33.7
**BT18**	1.86937354	III	AOA	17.4	1187.2	23.2
**BT55**	2.37788373	III	AOA	4.3	351.0	59.0
**BT126**	2.55342919	IV	GBM	10.9	362.0	77.5
**BT155**	3.32685118	III	AOA	27.6	2101.7	22.0
**BT131**	3.32812272	III	AOA	53.3	2925.0	30.8
**BT141**	4.45244737	II	O	85.8	3643.0	37.4
**BT22**	5.50056448	III	AOA	16.8	843.3	56.4
**BT55**	7.22385196	III	AOA	4.3	351.0	59.0
**BT116**	7.58670253	III	AOA	3.9	150.4	75.6
**BT136**	7.6550655	IV	GBM	1.4	72.2	62.5
**BT77**	10.2550999	III	AOA	58.4	2020.5	36.5
**BT34**	12.0645712	III	AOA	68.9	2685.3	56.1

Table containing Freiburg patients information including DNMT1 expression data by qRT-PCR (S18-normalized), overall survival (OS in days), progression-free survival (PFS in days) and age in years.
